# Comparison of The Effects of Cognitive Therapy and Logotherapy on Iranian Elderly People’s General Health

**DOI:** 10.30476/ijcbnm.2021.88217.1497

**Published:** 2021-10

**Authors:** Ali Ravari, Tayebeh Mirzaei, Fatemeh Hosieni, Elham Hassanshahi

**Affiliations:** 1 Deptartment of Medical Surgical Nursing, School of Nursing and Midwifery, Geriatric Care Research Center, Rafsanjan University of Medical Sciences, Rafsanjan, Iran; 2 Geriatric Care Research Center, Rafsanjan University of Medical Sciences, Rafsanjan, Iran

**Keywords:** Cognitive therapy, Frail elderly, Psychotherapy

## Abstract

**Background:**

Mental health promotion programs in the elderly are important. The main objective of the present study was to compare the effects of cognitive therapy and logotherapy on the general health of elderly people who referred to health centers.

**Methods:**

This is a quasi-experimental study with pretest-posttest design using a control group. A sample of 90 elderly people was recruited from health centers located in Rafsanjan, between April 2017 and June 2017. 30 participants were considered in each group, separately (cognitive therapy (N=30), logotherapy (N=30), and control (N=30)). Cognitive therapy and logotherapy programs were implemented in eight 90-minute sessions, while people in the control group received neither cognitive therapy nor logotherapy. The demographic questionnaire was used at baseline. The 28-item General Health Questionnaire (GHQ-28) was used in two steps of pre-test and post-test. The study data were analyzed through independent t-test, Chi-square, one-way ANOVA, and the Tukey’s post hoc tests; SPSS 18 was used to analyze the data, and P<0.05 was considered as significant.

**Results:**

The mean GHQ-28 total scores before the intervention in the cognitive therapy and logotherapy groups and control group were 52.53±2.55, 52.63±5.64, and 52.26±4.09, respectively, which were not significantly different (P=0.94). However, after the intervention these scores were 41.60±3.31, 40.46±3.97 and 51.93±4.22, respectively, and the difference between the two intervention groups and control group was statistically significant (P<0.001). There was no significant difference between the two intervention groups (P=0.49).

**Conclusions:**

Group cognitive therapy is as effective as logotherapy in improving the elderly people’s general health.

## INTRODUCTION

In most countries, the elderly population is growing faster than other age groups. ^1^ Statistics show that the global population growth rate is 1.7%, but this rate for the elderly population is 2.5%. ^2^ Therefore, it is anticipated that the elderly population in 2050 reach nearly 2 billion people. ^3^ A significant increase in the elderly population in exposure to this aging phenomenon is a cause for concern. ^4^

Physiological changes in old age may increase the risk of disease in the elderly people. One of the most prevalent age-related health problems is mental disorders. ^5^ About one third of all elderly people who refer to health centers suffer from mental health problems. ^6^ Treatment of such problems necessitates medication therapy. ^7^ However, because of the abundance of medications used by elderly people, the greater probability of forgetfulness among them, and dysfunction of detoxifying bodily organs (such as the liver and kidneys), they are at greater risk of experiencing the side effects of medications. ^8^ Thus, non-pharmacological therapies are of paramount importance to the elderly people.

Previous studies have shown that social support has beneficial effects on the elderly people’s physical and mental health in that people with stronger social support perceive less psychological pressure, have greater self-confidence, and have greater longevity. ^9 , 10^ A study found that, compared with people who have limited communication with others, elderly people who join social networks and receive social support enjoy better mental and physical health. ^11^

One of the greatest sources of support for elderly people, particularly for those who live at private homes, is the health centers. ^12^ To protect and promote the mental health of the elderly people who live in private homes, health centers need to develop and implement mental health promotion programs. ^11^ A good strategy for supporting the elderly people is counseling; however, individual counseling is too costly. Hence, group therapy is used to improve counseling-related cost-effectiveness. ^13^ One study has indicated that group counseling is as effective as medication therapy and is associated with fewer side effects. ^14^ Another study found that group spiritual care can increase the self-efficacy of the family caregivers of Alzheimer’s disease. ^15^

Two most common group counseling techniques for elderly people are cognitive therapy and logotherapy. ^16^ Logotherapy is a technique in which a client is guided to find some meaning in life by using his/her philosophical, theoretical, and religious backgrounds. ^17^ Teamwork in logotherapy helps people resolve their problems, overcome their grief and agony, and give meaning to their lives. ^18^

Cognitive therapy is another group counseling technique which comprises a set of methods for changing the individuals’ negative attitudes toward themselves, the world, and the future. ^19^ It helps people substitute old ideas and beliefs with new ones, focus on positive aspects of situations and events, and expand their capabilities for changing and correcting their depressive thoughts. Accordingly, it changes the individuals’ interpretations of the phenomena. ^20^

The purpose of cognitive therapy is to examine automatic negative thoughts; find connections between those thoughts, feelings and behaviors; change erroneous arguments into logical arguments; and help the patients recognize and change erroneous ideas. ^21^ Logotherapy tries to make the person aware of his/her responsibility and leaves it up to the individual to choose for whom and what he/she is responsible. By applying logotherapy, the patient learns to stop worrying and pay attention to something else. ^18^

A study has shown the effectiveness of cognitive therapy and logotherapy in nursing homes located in our country, Iran, without making any comparison between their effects. ^13^ Another study has shown the effectiveness of cognitive therapy and logotherapy in male nursing home residents’ depression in our country, Iran, with making comparison between their effects. ^17^

According to the fact that counseling (especially group therapy) is one of the appropriate strategies to support the elderly and given that in our knowledge the effects of cognitive therapy and logotherapy on all scales of general health of the elderly in Iran have not been compared; also, due to the characteristics and potentials of the cognitive therapy and logotherapy methods, this study aimed to compare the effects of cognitive therapy and logotherapy on Iranian elderly people’s general health.

## MATERIALS AND METHODS

This is a quasi-experimental study in the form of pretest-posttest using a control group.

In the current study, 90 elderly people who referred to the health center affiliated to Rafsanjan University of Medical Sciences, Rafsanjan, Iran between April 2017 and June 2017 were recruited. Inclusion criteria for this study were Iranian nationality, age of 60-80 years, ability to understand and speak Persian, ability to perform daily activities, and lack of a history of known psychosis and depression (a Geriatric Depression Scale (GDS-15) score of below 5). Yesavage et al. in 1982 developed GDS as a 30-item yes/no questionnaire and in 1983 they reported validity and reliability of the questionnaire. ^22 , 23^ GDS-15 is a short form of GDS-30 item which was developed by Sheikh and Yesavage in 1986 ^24^ although they showed that scores on the GDS-15 had a high correlation with the original GDS-30 (r=0.84). ^24^ Boey in 2000 conducted a study to show the psychometric properties of the GDS-15. In this study, the reliability of the GDS-15 in terms of internal consistency (Cronbach α=0.80) and test-retest reliability (r=0.73) was satisfactory. Also, concurrent validity of the GDS-15 was demonstrated by its differential relationships with negative and positive affect (r=0.43, r=-.019). ^25^ GDS-15 consists of 15 yes/no questions and each question is scored as either 0 or 1 points. Scores of 0-5, 6-10, and 11-15 are seen as normal range, moderate depression, and severe depression, respectively. It is noteworthy that 10 out of the 15 items indicated the absence of depression when answered negatively, while the rest (items: 1, 5, 7, 11, 13,) indicated lack of depression when answered positively. ^24^ We employed the Persian version of the GDS-15. It was developed by translation and back-translation and its reliability was measured by Malakouti et al. in 2006 using test-retest (r=0.58). ^26^ Exclusion criteria included social and family crises during the study, use of psychotropic drugs, and grief experience during the study. Participation in the study was voluntary and the participating elderly people could withdraw from the study at their own preferences.

To estimate the sample size, we used type one (α) and type two errors (β) of 0.05 and 0.10 (power=90%), d=8, σ=7.55 based on a previous study, ^6^ using the following formula 

$n=\frac{2{\left(z\frac{\mathrm{1-\alpha}}{2}+z_{\mathrm{1-\beta}}\right)}^{2}\sigma^{2}}{d^{2}}$

$n=\frac{2{\left(1.96+1.29\right)}^{2}7.55^{2}}{8^{2}}=19$

The minimum required sample for each group was estimated to be 19 people, while for three groups in each group n√3-1=19×1.4=26 people were obtained and for added Assurance, 30 people were considered for each group. 

Initially, two health centers were selected through simple cluster random sampling from all 29 health centers located in Rafsanjan city; then, we referred to the Medical Records unit of the study setting) because of availability( and recruited 90 elderly medical records randomly. Next, 30 participants were considered in the intervention group under the treatment of the cognitive therapy. In addition, 30 participants were in the logotherapy group. The control group consisted of 30 elderly people, too. Then, the elderly people were phoned and invited to participate in the study; after giving their written informed consent, they were invited to attend a session, on a predetermined date to provide detailed information about the study process. If an elderly person refused to participate or was inaccessible, another suitable one was selected, as mentioned previously. 

Participants in the experimental groups received either cognitive therapy or logotherapy in fifteen-person small groups and in eight 90-minute group counseling sessions which were held and managed by a Master’s degree holder in psychology with the necessary certificates for holding cognitive therapy and logotherapy classes. The cognitive therapy and logotherapy programs were developed based on textbooks. ^18 , 27^ The members of the cognitive therapy group passed the sessions of cognitive therapy twice weekly for 4 weeks (Table 1). Additionally, in each session, the contents of the previous sessions were reviewed, homework assignments were checked, and assignments for the next session were determined. The members of the logotherapy group passed the sessions of the logotherapy twice weekly for 4 weeks (Table 2). Members of the control group did not receive any group counseling intervention.

**Table 1:** The content of the sessions in the cognitive therapy group

Number of sessions	Content of sessions
First session	Introducing members, explaining about the goals and the expected outcomes of the programs, determining the length and the place of the sessions based on their preferences, asking about the nature and the causes of their current health problems, problem-related thoughts, and their life experiences
Second session	Training the three domains of thought, behavior, and physiology; the chain of A-B-C; and the cognitive behavioral therapy model;
Third Session	Recognizing thoughts and assessing their processes
Fourth session	Changing the beliefs and assessing their relationships with emotions
Fifth session	Assessing the two main factors that affect self-esteem which include great expectations and comparison of self with others (i.e. real and ideal selves)
Sixth session	Self-control education, problem solving, rational modification of behaviors, and rational analysis of them
Seventh session	Exercising behavior modification, strengthening positive thoughts, and correcting negative ones
Eighth session	Asking the elderly to summarize the contents of the sessions; and asking the participants to answer the questionnaire

**Table 2:** The content of sessions in logotherapy group

Number of sessions	Content of sessions
First session	Introducing members, explaining about the goals and the expected outcomes of the programs, determining the length and the place of the sessions based on their preferences, problem-related thoughts, asking the elderly to do the homework and thinking about it, commenting about it at the next session
Second session	Getting familiar with ‘meaning’, its sources, and how to find it through love
Third Session	Finding meaning in agony through values as well as finding enjoyment in doing activities
Fourth session	Finding meaning through empirical values
Fifth session	Enabling the participants to go beyond self and laugh at problems
Sixth session	Finding meaning through referring to one’s own past and encouraging the participants to accept greater responsibility towards finding meaning in the present time
Seventh session	Reviewing the sources of meaning and helping the participants use such sources in their daily lives
Eighth session	Asking the elderly to summarize the contents of the sessions; and asking the participants to answer the questionnaire

The time and place of training for the intervention groups were fixed. Training classes were held in the special room for the therapy group of the health center on Sundays and Wednesdays (at 10-11:30 AM) for the cognitive therapy groups and on Saturdays and Tuesdays (at 10-11:30 AM) for the logotherapy groups. An hour before the start of class, a call was made to all the elderly to remind them to attend the class. The next day of each class, homework was reminded during the phone call.

The study instruments were the demographic questionnaire which included items regarding the participants’ age, gender, marital status (single in this study means not married, dead spouse, and divorced), education level, and the 28-item General Health Questionnaire (GHQ-28). This is a self-administered questionnaire, designed for detecting non-psychotic psychiatric illnesses in primary health care by Goldberg in 1972 as a 60-item scale. ^28^ It was later shortened into several versions. GHQ-28 was designed using the implementation of factor analysis method of the original GHQ-60, and it was developed by Goldberg et al. in 1979. Varimax rotation of 4 factors accounted for 59% of total variance. ^29^ Goldberg et al. in 1997 tested the GHQ–28 against clinical ratings in 15 different sites around the world and reported a good validity of the questionnaire again. ^30^ Robinson et al. in 1982 reported the high reliability of GHQ-28 (r=0.90), using test-retest. ^31^ It contains four seven-item subscales of somatic symptoms (items 1–7), anxiety and sleep disorder (items 8–14), social dysfunction (items 15–21), and depression symptoms (items 22–28). ^29^ The possible responses to each item are not at all (scored 0), no more than usual (scored 1), and rather more than usual (scored 2), and much more than usual (scored 3). The lowest and highest total GHQ-28 scores and subscale scores were from 0- 84 and 0-21, respectively. Subscale scores of greater than 9 or a total GHQ-28 score of greater than 22 reflect general health problems. ^29^ We employed the Persian version of the GHQ-28. It was developed by translation and back-translation and its reliability coefficients were measured by Taghavi in 2002, using three different methods of test retest (r=0.70), split half (r=0.93), and Cronbach alpha (α=0.90); its validity was confirmed using concurrent validity, measured by Middlesex Hospital Questionnaire (r=0.55). Factor analysis of the questionnaire using varimax rotation based on Scree test covered over 50 percent of the total variance. ^32^ The study participants were invited to complete the GHQ-28 both before and immediately after the end of the study interventions. It is noteworthy that a trained nurse was in charge of calling the participants, following up for 4 weeks, recalling of meeting times, and helping illiterate participants with answering questionnaires.

The study was approved by the Ethics Committee of Rafsanjan University of Medical Sciences (IR.RUMS.REC.1394.110). We provided detailed information about the aim and the flow of the study to the participants and ensured them that withdrawal from the study was voluntary. Moreover, we assured them of the confidentiality of their information. The data collector and the person who performed the analysis were not aware of the methods.

The Kolmogorov-Smirnov test was used to examine the normal distribution of variables. One-way analysis of variance (ANOVA) was used to detect the differences between the groups and paired t test to detect the differences within the groups. Chi-square test was used for comparison of categorical variables. To determine the effects of the intervention, we used the Tukey’s post hoc tests. P values less than 0.05 were considered statistically significant. All statistical analyses were performed using the Statistical Package for Social Science version 18 (SPSS Inc., Chicago, Illinois, USA).

## RESULTS

Chi-square test showed that the study groups did not differ significantly regarding demographic variables (P>0.05; Table 3). The mean age of the participants was 70.02±6.74 years. The One-way ANOVA showed that there was no significant difference between the age of the control (68.63±5.48 years), cognitive therapy (71.43±6.42 years), and logotherapy (70.00±6.74 years) groups (P=0.27).

**Table 3:** Demographic characteristics of the participants in experimental and control groups

Variable	Cognitive Therapy	Logotherapy	Control	*P value
	N (%)	N (%)	N (%)	
Gender				
Male	7 (23.30)	9 (30)	15 (50)	0.07
Female	23 (76.70)	21 (70)	15 50)	
Education				
Literate	4 (13.30)	5 (16.60)	7 (23.30)	0.58
Illiterate	26 (86.70)	25 (83.40)	23 (76.70)	
Marital status				
Single	10 (33.30)	10 (20)	10 (33.30)	0.42
Married	20 (66.70)	20 (80)	20 (66.70)	

* Chi square test

Before the intervention, the total GHQ-28 score among the three study groups was not significantly different (P=0.94; Table 4). The results of the one-way ANOVA showed that, except for the significant difference of pretest score of the depression symptoms subscale (P=0.03), the three study groups did not differ significantly from each other concerning the pretest scores of the subscales (P>0.05; Table 4).

**Table 4:** Comparison of the study groups regarding the total score of 28-item General Health Questionnaire and the scores of its subscales

GHQ-28^a^ and subscales scores	Before intervention	After Intervention	Mean Difference	Within Groups
	Mean±SD	Mean±SD		P value**
Somatic symptoms				
Control	14.06±2.42	13.86±2.44	-0.20±1.47	0.46
Cognitive therapy	13.86±1.77	11.16±1.36	-2.70±1.53	<0.001
Logotherapy	13.73±2.99	10.93±2.16	-2.80±2.55	<0.001
Between Groups	0.14	<0.001		
*P value				
Anxiety and sleep disorder				
Control	14.16±2.00	14.06±2.13	-0.10±1.29	0.42
Cognitive therapy	14.60±1.92	10.13±1.90	-4.46±2.06	<0.001
Logotherapy	13.33±2.49	9.40±2.35	-3.93±2.59	<0.001
Between Groups	0.07	<0.001		
*P value				
Social dysfunction				
Control	15.40±2.51	14.86±2.41	-0.53±1.75	1.66
Cognitive therapy	15.66±1.62	13.23±1.88	-2.43±1.99	<0.001
Logotherapy	14.90±2.26	12.70±1.74	-2.20±2.1	<0.001
Between Groups	0.38	<0.001		
*P value				
Depression symptoms				
Control	11.76±0.81	11.80±0.80	0.03±1.47	0.12
Cognitive therapy	11.83±2.45	7.00±1.10	-4.83±2.24	<0.001
Logotherapy	10.66±2.32	7.43±0.89	-3.23±2.32	<0.001
Between Groups	0.03	<0.001		
*P value				
Total				
Control	52.26±4.09	51.93±4.22	-0.33±4.96	0.71
Cognitive therapy	52.53±2.55	41.60±3.31	-10.93±3.80	<0.001
Logotherapy	52.63±5.64	40.46±3.97	-12.16±5.07	<0.001
Between Groups	0.94	<0.001		
*P value				

a 28-item General Health Questionnaire;

* One-way ANOVA;

** Paired t-test

The mean total GHQ-28 score before the intervention was not significantly different among the control and intervention groups (cognitive therapy group (P=0.96) and logotherapy group (P=0.94; Table 5). Also, the difference between the two intervention groups, according to the total GHQ-28 score before the intervention, was not statistically significant (P=0.99; Table 5). The eight session intervention showed an improvement in total GHQ-28 score in the intervention groups compared to the controls. The difference between the two intervention and control group was statistically significant (P=0.001). The Tukey’s post hoc test did not show a significant difference between the two intervention groups (P=0.49; Table 5).

**Table 5:** Comparison of mean 28-item General Health Questionnaire scores in the study groups relative to each other before and after the intervention

Mean GHQ-28^a^ Score	Group	Mean Difference	*P value
Before	Control		
	Cognitive Therapy	-0.26	0.96
	Logotherapy	-0.36	0.94
	Cognitive Therapy		
	Control	0.26	0.96
	Logotherapy	-0.10	0.99
	Logotherapy		
	Control	0.36	0.94
	Cognitive Therapy	0.10	0.99
After	Control		
	Cognitive Therapy	10.33	0.001
	Logotherapy	11.46	0.001
	Cognitive Therapy		
	Control	-10.33	0.001
	Logotherapy	1.13	0.49
	Logotherapy		
	Control	-11.46	0.001
	Cognitive Therapy	-1.13	0.49

a 28-item General Health Questionnaire;

* Tukey’s post hoc test

After the intervention, the highest differences between the control and experimental groups were related to the depression symptoms (P<0.001), anxiety, and sleep disorders subscales (P<0.001) respectively, while the lowest difference was related to the social dysfunction subscale (P<0.001; Table 4).

Regarding the comparison of before and after the intervention and control groups, the findings of the study showed that in the control group, the mean GHQ-28 score and its subscales after the study did not change compared to before, and the paired t-test did not show a significant difference (P>0.05). However, in both intervention groups, the mean GHQ-28 score and all subscales decreased after the intervention and the independent t-test showed a significant difference (P<0.05; Table 4).

## DISCUSSION

The results of the present study showed that group logotherapy was effective in improving the general health of the elderly. The results of a study also showed that group logotherapy significantly improved the quality of life and the general health of female nursing home residents. ^13^ Moreover, logotherapy has been reported by previous studies to have positive effects on the elderly men’s depression and elderly women’s general health. ^13 , 17^ Our findings are consistent with those reported by previous studies, maybe due to the similarity in the population of the studies. However, the results of another study showed that although group logotherapy was effective in alleviating the elderly women’s anxiety, social dysfunction, and depression, it had no significant effect on their somatic symptoms. ^33^ They attributed this finding to the fact that in nursing homes, the elderly people are managed and treated in the same way as hospital settings; hence, nursing home residents tend to magnify their physical problems either intentionally or unintentionally in order to receive closer attention.

The findings of the present study also showed that cognitive therapy was effective in improving the elderly people’s general health. Cognitive therapy has been reported to exert positive effects on physical and functional parameters, psychotic symptoms, and depression. ^17 , 34 , 35^ We also found that cognitive therapy significantly affected all subscales of the GHQ-28. However, the results of a study showed the effectiveness of cognitive therapy in chronic bodily pain. ^36^ The consistency of our findings with those reported by previous studies may be due to the fact that the study population was at the old age in all studies. It seems that cognitive therapy helps the elderly people understand that problems are not unique to them and other elderly people also have the same experiences and problems. Accordingly, it alleviates feelings such as loneliness, self-blame, shame, and guilt and enables them to exchange their solutions to problems and their coping skills. Such shared experiences and communications boost their self-esteem and self-concept and help them overcome the difficulties of their lives. However, the results of another study showed that cognitive therapy intervention has no effect on reducing depression. ^37^ Probably, the difference in the age range of the statistical population and the history of depression can be important reasons for the difference in the results of our study and the previous study. 

Furthermore, the findings of the present study revealed that logotherapy was as effective as cognitive therapy in enhancing the elderly people’s general health. However, the results of a research showed that although both logotherapy and cognitive therapy were effective in alleviating male nursing home residents’ depression, the effects of cognitive therapy were stronger than logotherapy. ^17^ This contradiction between our findings and those reported by previous study may be due to the differences in the settings, interventions, and sample sizes of the studies. The findings of the present study showed that both logotherapy and cognitive therapies were effective in improving anxiety and sleep disorder, social dysfunction, somatic symptoms and depressive symptoms in the elderly, and the highest and lowest improvements were related to depression symptoms and social dysfunction, respectively. The results of a study showed that both logotherapy and cognitive therapy were effective in alleviating the nursing home male residents’ depression. ^17^ The strength of our study compared to this study was the study of the GHQ-28 subscales in the elderly.

As most elderly people believe that no one needs them and their services anymore, they may experience deviations from physical and mental health. ^33^ Besides, according to the Theory of Activity which deals with the positive relationship of social activities with life satisfaction, participation in group logotherapy and cognitive therapy sessions is a social activity which can promote the elderly people’s general health and help them gain control over their lives through changing their attitudes and behaviors. ^38^ In general, participation in group counseling sessions helps the elderly people attract the attention they need from group members. Also, according to the results of this study, one of the two methods can be used as a non-pharmacological treatment option to improve the general health of the elderly according to their choice. 

In this study, participants in cognitive therapy or logotherapy training groups received only eight 90-minute sessions of group counseling, and the present study was conducted over a period of 4 weeks to compare the effects of cognitive therapy and logotherapy on Iranian elderly people. Thus, one of its limitations was the short-term group counseling and follow-up of the elderly. The study of two methods of group counseling with the inclusion of a control group is a strength of the study design. 

## CONCLUSION

The findings of the current study show that group cognitive therapy is as effective as logotherapy in improving elderly people’s general health. Therefore, cognitive therapy and logotherapy can be used as effective non-pharmacological interventions for promoting the elderly people’ general health. It is recommended that this study should be repeated with other psychological therapies such as problem-solving skills and stress management in the elderly with other cognitive disorders such as depression or be performed on other age groups; further studies are required to investigate whether these early beneficial effects persist over longer durations or not.
